# Single or Combined Supplementation of *Rhodotorula* Yeast Culture and *Bacillus Subtilis* Enhances Intestinal Barrier Function in Yellow-Feathered Broilers

**DOI:** 10.3390/vetsci12060558

**Published:** 2025-06-06

**Authors:** Xiangtan Su, Ke Wang, Yeqing Liu, Xinyu Lu, Meiru Chen, Jianlong Dang, Gaowei Zhang, Guang Yang, Aiqin Gao, Yuanqing Xu

**Affiliations:** College of Animal Science, Inner Mongolia Agricultural University, Hohhot 010018, China; fs646464@163.com (X.S.); wangke99658@163.com (K.W.); jiangseruyan@163.com (Y.L.); 15134666337@163.com (X.L.); jjjjtmmjt@163.com (M.C.); perter1733@formail.com (J.D.); 17637324277@163.com (G.Z.); yangguang_94@126.com (G.Y.)

**Keywords:** yellow-feathered broilers, *Rhodotorula* yeast culture, *Bacillus subtilis*, intestinal immunity, intestinal barrier function

## Abstract

This study aimed to investigate the effects of dietary supplementation with *Rhodotorula* yeast cultures (RYC) and *Bacillus subtilis* (BS), alone or in combination, on growth performance and intestinal barrier function in yellow-feathered broilers. A 2 × 2 factorial interaction study was conducted, in which a total of 192 one-day-old yellow-feathered broilers were randomly assigned into four treatment groups: CON group, fed a basal diet; BS group, supplemented with 5 × 10^9^ CFU/kg of BS; RYC group, supplemented with 5000 mg/kg of RYC; and RYC + BS group, supplemented with both. During the 56-day experimental period, body weight, average daily feed intake, average daily gain, and feed conversion ratio were not significantly affected by RYC, BS, or their interaction (*p* > 0.05). In the jejunum, the villus height-to-crypt depth ratio was significantly increased by the interaction of RYC and BS (*p* < 0.05). mRNA expression of tight junction proteins (*JAM2*, *TJP1*) was significantly upregulated by BS alone (*p* < 0.05), but this effect was diminished when RYC and BS were combined, indicating an antagonistic interaction between the two supplements. Mucin-2 (*MUC2*) expression was significantly increased by RYC or BS alone (*p* < 0.05). In immune function analysis, IgM levels were significantly increased by RYC alone but decreased when RYC and BS were combined (*p* < 0.05), further highlighting their antagonistic interaction. BS supplementation significantly increased IgG and pro-inflammatory gene expression (*TNFA*, *IL1B*, and *NFKB1*) (*p* < 0.05), while RYC supplementation reduced IFN-γ and increased anti-inflammatory gene expression (*IL10* and *MyD88*). Cecal microbial analysis revealed increased abundance of *g_Bacillus* in the BS group, *g_norank_f__norank_o__Clostridia_UCG-014* in the RYC group, and *g_norank_f__norank_o__norank_c_Clostridia* in the BS + RYC group were significantly increased compared to the CON group (*p* < 0.05). These results suggest that RYC and BS, as dietary supplements, may enhance intestinal health and barrier function in yellow-feathered broilers with minimal effects on growth performance. However, the antagonistic interactions between RYC and BS in modulating immune responses and tight junction protein expression highlight the need for careful consideration when combining these supplements in poultry nutrition strategies.

## Simple Summary

Yellow-feathered broilers, a distinctive breed in China, are more susceptible to immune dysfunction and intestinal diseases due to intensive farming practices. To address these challenges, enhancing the intestinal barrier function is a critical strategy. This study investigated the effects of *Rhodotorula* yeast culture (RYC) and *Bacillus subtilis* (BS), either alone or in combination, on the intestinal health of yellow-feathered broilers. RYC has been shown to alleviate immune stress and strengthen intestinal barrier function, while BS can regulate intestinal microbiota and maintain gut health. The experimental results revealed that the combination of RYC and BS improved jejunal structure, as evidenced by an increased villus height-to-crypt depth (VH/CD) ratio. However, the two supplements exhibited antagonistic interactions in modulating immune responses and the expression of tight junction proteins. Despite these antagonistic effects, the study highlighted the potential of RYC and BS to enhance intestinal barrier function and modulate immune activity in yellow-feathered broilers. These findings provide valuable insights into the scientific application of RYC and BS as dietary supplements, offering strategies to improve gut health and overall well-being in poultry farming.

## 1. Introduction

Yellow-feathered broilers, a distinctive breed in China, are highly valued for their distinctive flavor profiles and superior nutritional composition [1]. This growing consumer preference has driven their expanding market share in China’s meat sector [2]. However, modern intensive breeding practices aimed at accelerating growth rates—particularly through high-fat diets—impose substantial intestinal stress, compromising barrier integrity in broilers [3]. Such barrier dysfunction can trigger a cascade of metabolic dysregulation, manifesting as suppressed growth metrics, amplified stress susceptibility, and impaired immunocompetence [4]. These challenges are exacerbated by the breed’s extended production cycle, necessitating more rigorous gut health management compared to commercial broiler strains [5].

The gastrointestinal tract serves as the primary interface between external environments and internal homeostasis, with its multilayered barrier system providing a critical defense against luminal pathogens and antigens [6]. The intestinal barrier system consists of a tightly connected epithelial cell layer, tight junction protein (TJP) expression, mucus secretion, intestinal immune cells, and the resident symbiotic microbiota [7]. The system is categorized into four distinct types based on their functions: the physical barrier, the chemical barrier, the immune barrier, and the microbial barrier. TJP, specialized structures situated between intestinal epithelial cells, function as a critical regulatory barrier that governs paracellular permeability, maintaining gut barrier integrity [8].

Emerging evidence shows that synbiotic interventions (combined prebiotics and probiotics) can optimize the intestinal ecosystem by reshaping microbial composition, enhancing α-diversity, and competitively excluding enteropathogens [9,10]. Within this paradigm, yeast cultures—a class of multifunctional probiotic preparations—deliver a bioactive matrix comprising fermentation substrates, microbial proteins, yeast metabolites, and cell wall components like β-glucans and mannooligosaccharides [11]. Notably, yeast-derived components have been shown to increase the ileal villus height-to-crypt depth (VH/CD) ratio in broilers [12]. Yeast cell walls also enhance intestinal barrier integrity in broilers through the upregulation of TJP gene expression, thereby improving gut health [13,14]. Additionally, dietary yeast culture supplementation has been found to modulate the composition and diversity of the cecal microbiota in broilers [11]. Beyond structural benefits, red yeast enhances gastrointestinal health in poultry by adsorbing mycotoxins, reducing toxin-induced intestinal dysfunction [11,15]. Importantly, studies have shown that *Rhodotorula* yeast culture (RYC) is rich in metabolites such as carotenoids, which provide significant benefits in reducing immune stress and enhancing intestinal barrier function [16].

*Bacillus subtilis* (BS), a prominent member of the animal intestinal microbiota, produces bioactive compounds such as lipopeptides, surfactants, and bacteriocins, which can be used as a viable alternative to traditional feed antibiotics [17,18]. Beyond its antimicrobial effects, BS enhances intestinal health in broilers by improving VH/CD, upregulating TJ expression, and reducing pathogenic colonization in the cecum, thereby synergistically improving gut barrier integrity and microbiota homeostasis [19,20]. Additionally, BS facilitates nutrient release, modulates digestive enzyme activity, and optimizes microbial community structure, further supporting intestinal function [21,22].

Recent studies have demonstrated that combining dietary supplementation with BS and *Saccharomyces cerevisiae* can significantly improve growth performance in *Salmonella Typhimurium*-challenged broilers, primarily by enhancing intestinal morphology [23]. Further research suggests that BS achieves its maximal viable biomass when co-administered with yeast, indicating a potential microbial synergy [24].

Despite these findings, most studies have focused on the isolated application of yeast products or BS, leaving a critical gap in understanding their combined effects—particularly in yellow-feathered broilers. Specifically, systematic studies evaluating the impact of RYC and BS cosupplementation on intestinal barrier function are lacking. This study hypothesizes that the synergistic use of RYC and BS will enhance intestinal barrier function through complementary mechanisms: RYC primarily supports intestinal morphology via nutrient provision, while BS exerts its benefits through cecal microbiota modulation.

## 2. Materials and Methods

This study was conducted in accordance with the ethical guidelines and protocols of the People’s Republic of China. All experimental procedures were assessed and approved by the Animal Care and Use Committee at Inner Mongolia Agricultural University (Hohhot, China; Approval No. NND2024124).

### 2.1. Feed Additives

The *Rhodotorula* yeast culture (β-glucan ≥ 1.45 mg/g, mannan oligosaccharides ≥ 0.33 mg/g, carotenoids ≥ 1.60 mg/g) was provided by the State Key Laboratory of Animal Nutrition at the Beijing Institute of Animal Husbandry and Veterinary Medicine, Chinese Academy of Agricultural Sciences (Beijing, China). The RYC was produced by fermenting *Rhodotorula mucilaginosa* in a liquid broth with soybean meal as the solid substrate. The final product contained yeast cell walls, cellular metabolites, and residual medium components [25,26]. The *Bacillus subtilis* was provided by Beihai Yiqiang Biotechnology Co., Ltd. (Beihai, China) and had a viable count of 1 × 10^11^ CFU/g. Both the RYC and BS were used in powder form for this study.

The additives were incorporated into the feed in a three-stage mixing process. In the initial mixing stage, the probiotics (RYC or BS) were thoroughly blended with 100 g of mixed feed according to the experimental design. During the secondary mixing stage, this 100 g mixture was then combined with 900 g of standard feed, producing 1 kg of supplemented feed. In the final dilution stage, the 1 kg supplemented feed was mixed with 9 kg of standard feed, resulting in 10 kg of intermediate feed. For complete feed production, the 10 kg intermediate feed was blended with 30 kg of standard feed, yielding 40 kg of final feed. To ensure uniform distribution, the feed was mixed thoroughly at each stage to prevent ingredient separation [27].

### 2.2. Animals, Research Protocol, and Nutritional Regimens

A total of 192 one-day-old yellow-feathered broilers with similar initial body weights (40.00 ± 0.64 g) were randomly assigned to four treatment groups. Each group consisted of six replicate pens, with eight chicks per pen (four males and four females) to ensure sex balance. Based on previous research [28,29], the optimal inclusion levels of RYC and BS in the broiler diets were determined. The experiment was designed as a 2 × 2 factorial interaction study to evaluate the effects of RYC and BS, alone or in combination, on yellow-feathered broilers. The four experimental groups were as follows: Control group (basal diet only), BS group (basal diet + 5 × 10^9^ CFU/kg BS), RYC group (basal diet + 5000 mg/kg RYC), and BS + RYC group (basal diet + both additives: 5 × 10^9^ CFU/kg BS + 5000 mg/kg of RYC). The basal diet was formulated according to the Nutrition Requirement for Yellow-Feather Broilers (NY/T 3645-2020) [30]. The detailed composition and nutritional profile of the basal diet are provided in Table 1.

### 2.3. Assessment of Growth Performance and Acquisition of Samples

Feed intake and residual feed were recorded daily throughout the trial. Body weights were measured at two time points: the initial weight was recorded after a 2-h fasting period posthatching, and the final weight was measured at 56 days following a 12-h fast to ensure an empty gastrointestinal tract. Average daily gain (ADG), average daily feed intake (ADFI), and feed-to-gain ratio (F/G) were calculated per replicate using the following formulas:

ADG = (final body weight − initial body weight)/trial duration (days)

ADFI = feed intake/trial duration (days)

F/G = ADFI/ADG

At 56 days, six broilers per group (three males and three females, representing average body weight per sex) were selected for sampling. Following the administration of anesthesia, the selected yellow-feathered broilers were euthanized via carotid artery transection. A longitudinal surgical section was made along the ventral median axis to access the coelomic compartment. The duodenum, jejunum, ileum, and cecum were sequentially removed. During sampling, the contents of the distal end of the right cecum were collected uniformly, and 1.5 mL of the contents were rapidly transferred into a 2 mL freezing tube and flash-frozen in liquid nitrogen. A 2 cm segment of intestinal tissue from the midileum was excised, and the luminal digesta were flushed with 4 °C normal saline. The tissue was then placed into a 2 mL cryogenic storage tube and flash-frozen in liquid nitrogen. Duodenal, jejunal, and ileal samples were taken from approximately 1 cm of tissue 3 cm from the pylorus, 5 cm proximal to the yolk sac diverticulum, and 10 cm proximal to the ileocecal junction, respectively, for morphometric analysis. The samples were fixed in a 4% paraformaldehyde solution. All procedures were conducted in accordance with the methods described by Liu et al. [31].

### 2.4. The Structural Characteristics of Enteric Tissue

Fixed intestinal tissue samples were processed through progressive ethanol dehydration, paraffin embedding, sectioning, and hematoxylin and eosin (H&E) staining. Morphometric measurements, including villus height (VH), crypt depth (CD), and the VH/CD ratio, were performed using a Nikon Eclipse E200 microscope (Tokyo, Japan) and Image View 4 software.

### 2.5. Intestinal Biomarkers

A 0.5 g sample of ileal tissue was placed in a freezing tube, and 4.5 mL of phosphate-buffered saline was added to prepare a 1:9 tissue homogenate. The homogenate was centrifuged at 3500 rpm for 15 min. After centrifugation, the supernatant was collected and stored for further analysis; the remaining steps were carried out strictly according to the instructions. The total protein concentration in the supernatant was measured using a commercial BCA assay kit (Nanjing Jiancheng Bioengineering Institute, Nanjing, China). This was done with the Bicinchoninic Acid Assay according to the manufacturer’s guidelines. Each protein concentration measurement was performed in three technical replicates to ensure precision with intra-assay CV < 5%, inter-assay CV < 8%. Assays for chicken tissue immunoglobulins (IgG, JYM0001Ch; IgM, JYM0060Ch; sIgA, JYM0036Ch) and cytokines (TNF-α, JYM0033Ch; IFN-γ, JYM0007Ch; IL-1β, JYM0041Ch; IL-6, JYM0028Ch; IL-10, JYM0040Ch) were performed using ELISA kits (Genome Biotechnology Co., Ltd., Wuhan, China). For ELISA assays, samples were analyzed in duplicate per the manufacturer’s recommendations, with intra-assay CV < 10%, inter-assay CV < 12%.

### 2.6. Real-Time qPCR Assay

RNA isolation was performed according to the manufacturer’s guidelines (Takara, Dalian, China). Briefly, 50 mg of tissue samples were mechanically homogenized in 1 mL of Trizol lysate using an FA-25D homogenizer (FLUKO, Shanghai, China). mRNA extraction was conducted following the method described by Ji et al. [32]. RNA concentration and purity were measured using an Implen P330 spectrophotometer (Implen, Munich, Germany).

For cDNA synthesis, the *Evo M-MLV* RT MIX KIT (Accurate Bio-technology Co., Ltd., Changsha, China) was used. The reaction mixture consisted of 2 μL 500 μg/μL RNA, 2 μL gDNA Clean Reaction Mix Ver.2, and 6 μL enzyme-free water, which was incubated in a PCR instrument (Labcycler, Senso Quest, Germany) for 2 min. Subsequently, 4 μL 5 × *Evo M-MLV* RT Reaction Mix Ver.2 and 6 μL enzyme-free water were added. The reaction conditions were as follows: 37 °C for 15 min, 85 °C for 5 s, and 4 °C for cooling.

The qPCR assay was conducted using the YBR Green Pro Taq HS qPCR Kit (Rox Plus; Accurate Biotechnology Co., Ltd., Changsha, China). The PCR reaction system included 2 μL of cDNA, 0.5 μL of F-strand primer, 0.5 μL of R-strand primer, 10 μL of 2 × SYBR Green Pro Taq HS Premix, and 7.2 μL of enzyme-free water. RT-qPCR was performed on a LightCycler 480 II instrument (Roche, Basel, Switzerland) with the following program: 95 °C for 30 s (1 cycle), followed by 40 cycles of 95 °C for 5 s and 60 °C for 30 s.

*β-actin* was used as an endogenous normalization control to standardize the experimental data. The relative expression levels of the following target genes were analyzed: *Occludin* (*OCLN*), *zonula occludin-1* (*TJP1*), *junctional adhesion molecule-2* (*JAM2*), *mucin-2* (*MUC2*), *interleukin-1beta* (*IL1B*), *interleukin-6* (*IL6*), *interleukin-10* (*IL10*), *tumor necrosis factor-alpha* (*TNFA*), *interferon-γ* (*IFNG*), *myeloid differentiation factor 88* (*MyD88*), *Toll-like receptor 4* (*TLR4*), and *nuclear transcription factor-κBp50* (*NFKB1*). The relative mRNA expression of the target gene was calculated using the 2^-∆∆Ct^ method [33]. Primer sequences are provided in Table 2.

### 2.7. Ileal Microbiota Analysis

Samples were transported on dry ice to Shanghai Meiji Biomedicals Co. (Shanghai, China) to ensure sample integrity. Total DNA was extracted from the cecal contents of 56-day-old yellow-feathered broilers using the Fast DNA™ Spin Kit (MP Biomedicals, Santa Ana, CA, USA). The V3–V4 variable region of the bacterial 16S ribosomal RNA was amplified using the following primer sequences: forward primer 341F (5′-ACTCCTACGGGAGGCAGCAG-3′) and reverse primer 806R (5′-GGACTACHVGGGTWTCTAAT-3′). After quantitative purification, the amplified sequences were sequenced on the Illumina Nextseq 2000 PE300 platform. Raw sequences were processed using QIIME software (version 1.9.1) and clustered into amplicon sequence variants (ASV) [34]. All raw sequencing datasets have been deposited in the NCBI Sequence Read Archive under BioProject PRJNA1199219.

### 2.8. Statistical Analysis

All data were analyzed using two-way ANOVA to evaluate the main effects of RYC and BS, as well as their interaction effects, with the replicates serving as the experimental unit. Mean comparisons were performed using Duncan’s multiple range test. Statistical computations were conducted using SPSS version 21 (IBM Corp., Armonk, NY, USA) [35]. For cecal microbial data, analyses were performed on the online Majorbio cloud platform (www.majorbio.com, accessed on 10 December 2024). These analyses included alpha diversity analysis, beta diversity analysis, bacterial abundance analysis, and linear discriminant analysis effect size (LEfSe). The data analysis process involved three main steps: filtering raw data, applying the UPARSE algorithm for clustering, and annotating species. A *p* < 0.05 was considered statistically significant, and a *p*-value between 0.05 and 0.10 was considered indicative of a trend.

## 3. Results

### 3.1. Effects of RYC and BS Feeding on the Growth Parameters of Yellow-Feathered Broilers

As shown in Table 3, during the 56-day experimental period, body weight, ADFI, ADG, and F/G were not significantly affected by the main effects of RYC, BS, and their interaction in yellow-feathered broilers (*p* > 0.05).

### 3.2. Effects of Feeding RYC and BS on the Intestinal Morphology of Yellow-Feathered Broilers

**Figure 1:**
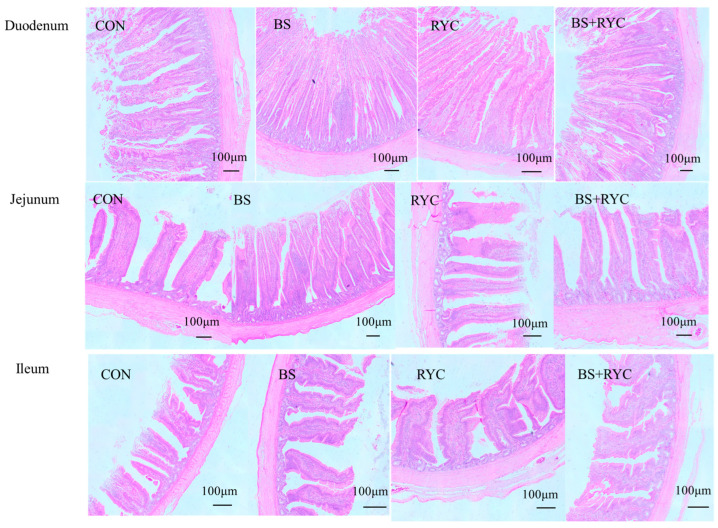
Optical microstructure of the intestine of yellow-feathered broilers supplemented with RYC and BS. Hematoxylin and eosin (H&E) staining results are presented for the duodenal, jejunal, and ileal intestinal segments. Control group (CON), basal diet: *Bacillus subtilis* group (BS), basal diet + 5 × 10^9^ CFU/kg BS; *Rhodotorula* yeast culture group (RYC), basal diet + 5000 mg/kg RYC; BS + RYC group (BS + RYC), basal diet + 5 × 10^9^ CFU/kg BS + 5000 mg/kg RYC. Figure 1 was examined at 40 × magnification and a scale bar = 100 μm.

In all experimental groups, epithelial cells were observed to be well-preserved, and normal mucosal architecture was identified in the duodenum, jejunum, and ileum, including intact mucosa, submucosa, and muscular layers (Figure 1). Notably, the intervillus space (the distance between adjacent intestinal villi) was analyzed and the following trends were observed: The narrowest intervillus spacing (indicating denser villus packing) was exhibited by the BS group, followed by the BS + RYC group, then the RYC group, with the widest spacing being recorded in the CON group. This trend was consistently observed in the duodenum, jejunum, and ileum.

As shown in Table 4, in the jejunum, the CD was significantly affected by the RYC × BS interaction; however, further analysis using Duncan’s multiple range test did not detect a significant effect (*p* < 0.05). In the jejunum, the VH/CD ratio was significantly affected by the RYC × BS interaction (*p* < 0.05). Further analysis using Duncan’s multiple range test revealed that the VH/CD ratio in the BS + RYC group was significantly higher than that in the other three groups (*p* < 0.05). The VH or VH/CD ratio in the duodenum, VH and CD in the jejunum, or VH, CD, and VH/CD ratio in the ileum were not significantly affected by the main effects of RYC, BS, and their interaction (*p* > 0.05).

### 3.3. Effects of Feeding RYC and BS on the Intestinal Physical and Chemical Barrier of Yellow-Feathered Broilers

As shown in Table 5, the mRNA expression of *OCLN*, *JAM2*, and *TJP1* was significantly affected by the RYC × BS interaction (*p* < 0.05). Further analysis using Duncan’s multiple range test on *OCLN* and *JAM2* revealed that the BS group was significantly higher than that in the other three groups (*p* < 0.05). Further analysis using Duncan’s multiple range test on *TJP1* revealed that the BS group was significantly higher than that in the CON and RYC groups (*p* < 0.05), and the BS + RYC group was significantly higher than that in the CON group (*p* < 0.05).

The mRNA expression of *MUC2* was not significantly affected by the RYC × BS interaction (*p* > 0.05). However, supplementation with either RYC or BS alone significantly increased the mRNA expression of *MUC2* (*p* < 0.05).

### 3.4. Effects of Feeding RYC and BS on the Intestinal Immune Barrier of Yellow-Feathered Broilers

#### 3.4.1. Effects of Feeding RYC and BS on the Ileal Cytokine and Immunoglobulin Contents of Yellow-Feathered Broilers

As shown in Table 6, IgM was significantly affected by the RYC × BS interaction (*p* < 0.05). Specifically, IgM levels were not affected by BS supplementation alone, were significantly increased by RYC supplementation alone, and were significantly decreased when both RYC and BS were supplemented together. Further analysis using Duncan’s multiple range test revealed that the RYC group had significantly higher IgM levels compared to the other three groups (*p* < 0.05). For IgG, BS supplementation significantly increased its content (*p* < 0.05), and there was no significant interaction between BS and RYC (*p* > 0.05). For IFN-γ, RYC supplementation significantly reduced its content (*p* < 0.05). For IL-1β, the RYC × BS interaction showed a trend toward significance (*p* = 0.052). BS supplementation significantly increased IL-1β concentration (*p* < 0.05), while RYC supplementation significantly decreased it (*p* < 0.05).

#### 3.4.2. Effects of Feeding RYC and BS on the Expression of Ileum Immunity-Related Genes of Yellow-Feathered Broilers

As shown in Table 7, the mRNA expression of *TNFA*, *IL1B*, and *NFΚB1* was significantly increased by BS supplementation (*p* < 0.05). For *IL10* and *MyD88*, RYC supplementation significantly increased their mRNA expression (*p* < 0.05). Additionally, the mRNA expression of *IL6* was significantly influenced by the RYC × BS interaction (*p* < 0.05). However, further analysis using Duncan’s multiple range test revealed no significant differences among the four groups (*p* = 0.117).

### 3.5. Effects of Feeding RYC and BS on the Microbiota of the Cecum of Yellow-Feathered Broilers

**Figure 2:**
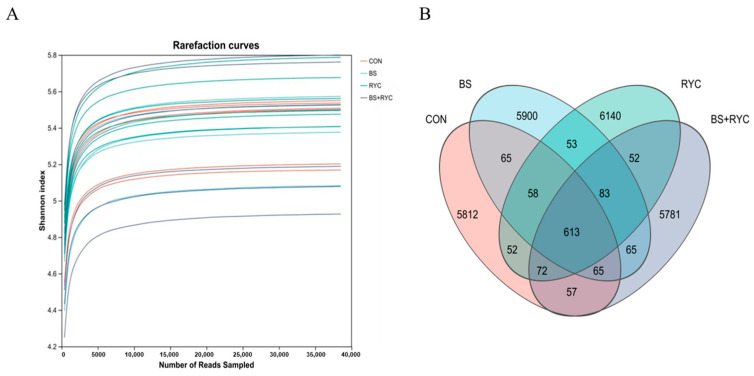
Effects of single or combined BS and RYC supplementation on microbial diversity in the cecum of yellow-feathered broilers. Rarefaction curves (**A**). Venn diagrams (ASV) (**B**). Control group (CON), basal diet; *Bacillus subtilis* group (BS), basal diet + 5 × 10^9^ CFU/kg BS; *Rhodotorula* yeast culture group (RYC), basal diet + 5000 mg/kg RYC; BS + RYC group (BS + RYC), basal diet + 5 × 10^9^ CFU/kg BS + 5000 mg/kg RYC. (*n* = 6).

A total of 38,510 clean reads were obtained from the cecum chyme samples. The rarefaction curves were observed to flatten out (Figure 2A), suggesting that a sufficient number of individual samples were collected from all four groups. Since the rarefaction curves were used to estimate the completeness of microbial community sampling, this result confirmed that the data were adequate for microbial community analysis.

To evaluate the effects of single or combined supplementation with BS and RYC, the count of shared and exclusive ASVs in the bacterial community of the cecum was analyzed using Venn diagrams. The results (Figure 2B) revealed that the four groups of cecum content samples collectively produced a total of 24,868 ASVs. Among these, 613 ASVs were identified as common across all four groups. The proportion of unique ASVs in each group was found to be 85.55% for the CON group, 85.48% for the BS group, 86.20% for the RYC group, and 85.16% for the BS + RYC group, respectively.

**Figure 3:**
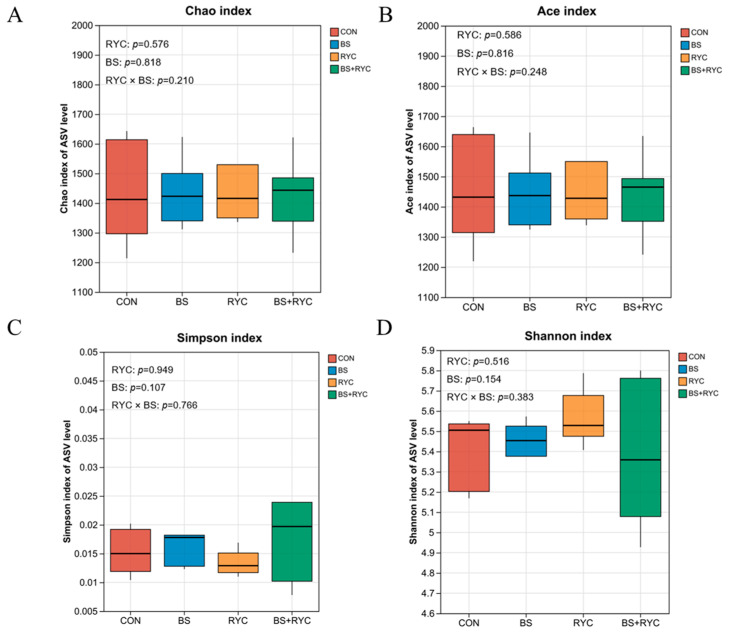
Effects of single or combined BS and RYC on the alpha diversity indices in the cecum of yellow-feathered broilers. Chao index at the ASV level (**A**); Ace index at the ASV level (**B**); Simpson index at the ASV level (**C**); Shannon index at the ASV level (**D**). Control group (CON), basal diet; *Bacillus subtilis* group (BS), basal diet + 5 × 10^9^ CFU/kg BS; *Rhodotorula* yeast culture group (RYC), basal diet + 5000 mg/kg RYC; BS + RYC group (BS + RYC), basal diet + 5 × 10^9^ CFU/kg BS + 5000 mg/kg RYC. All values are presented as mean ± SD (error bars), with the sample size indicated as *n* = 6. no statistically significant differences (*p* > 0.05) were detected between groups for these indices.

When assessing alpha diversity, the Chao index (Figure 3A), Ace index (Figure 3B), Simpson index (Figure 3C), or Shannon index (Figure 3D) was not significantly affected by the main effects of RYC, BS, and their interaction in yellow-feathered broilers (*p* > 0.05).

**Figure 4:**
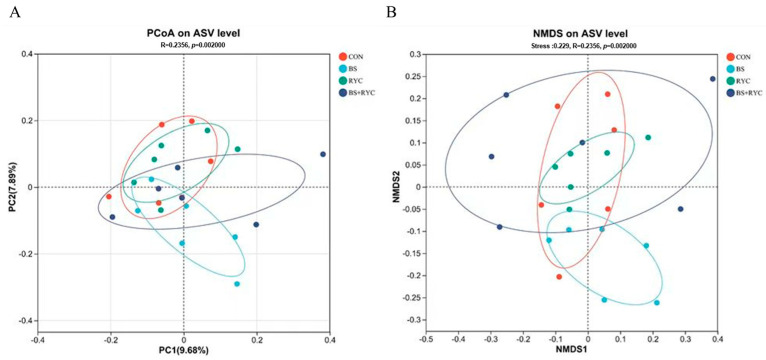
Effects of single or combined BS and RYC on the beta diversity indices in the cecum of yellow-feathered broilers. The assessment of cecal microbial communities was conducted using principal coordinate analysis (PCoA) (**A**) and nonmetric multidimensional scaling (NMDS) (**B**), employing the binary Curtis metric. Control group (CON), basal diet; *Bacillus subtilis* group (BS), basal diet + 5 × 10^9^ CFU/kg BS; *Rhodotorula* yeast culture group (RYC), basal diet + 5000 mg/kg RYC; BS + RYC group (BS + RYC), basal diet + 5 × 10^9^ CFU/kg BS + 5000 mg/kg RYC. (*n* = 6).

Beta diversity indices, analyzed through PCoA (Figure 4A) and NMDS (Figure 4B), revealed significant shifts in microbial communities at the ASV level (*R* = 0.236, *p* = 0.002) across the four groups.

**Figure 5:**
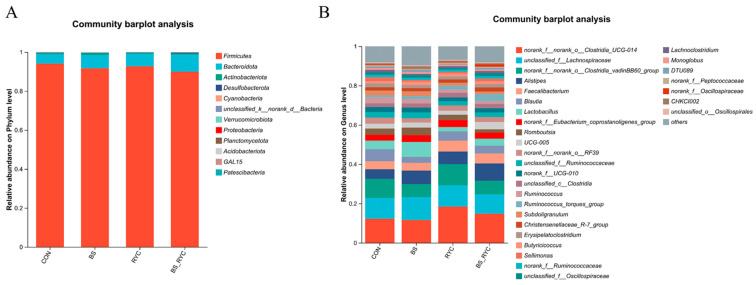
Effects of single or combined BS and RYC on the cecal microbiota of yellow-feathered broilers. The community composition of cecum microbiota at both the phylum and genus levels is represented as a percentage of abundance (**A**,**B**). Control group (CON), basal diet; *Bacillus subtilis* group (BS), basal diet + 5 × 10^9^ CFU/kg BS; *Rhodotorula* yeast culture group (RYC), basal diet + 5000 mg/kg RYC; BS + RYC group (BS + RYC), basal diet + 5 × 10^9^ CFU/kg BS + 5000 mg/kg RYC. (*n* = 6).

At the phylum level (Figure 5A), the cecal microbiota predominantly comprised *Firmicutes, Bacteroidota,* and *Actinobacteriota*, with *Firmicutes* accounting for over 90% of the total composition. At the genus level (Figure 5B), the dominant genera included *norank_f__norank_o__Clostridia_UCG-014*, *unclassified_f__Lachnospiraceae*, and *k_f__norank_o__Clostridia_vadinBB60_group*.

**Figure 6:**
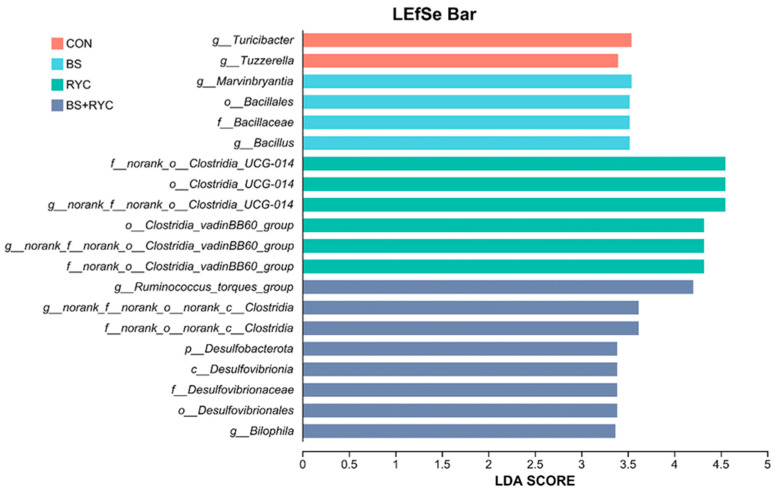
Effects of single or combined BS and RYC on cecal microbiota of yellow-feathered broilers. Linear discriminant analysis (LDA) discriminant histograms. Control group (CON), basal diet, *Bacillus subtilis* group (BS), basal diet + 5 × 10^9^ CFU/kg BS, *Rhodotorula* yeast culture group (RYC), basal diet + 5000 mg/kg RYC, BS + RYC group (BS + RYC), basal diet + 5 × 10^9^ CFU/kg BS + 5000 mg/kg RYC (*n* = 6).

As shown in Figure 6, LEfSe analysis (LDA score ≥ 3) revealed distinct microbial signatures across the treatment groups. In the CON group, the abundance of *g_Turicibacter* and *g_Tyzzerella* was significantly higher. The BS group exhibited an increased abundance of *g_Marvinbryantia*, *o_Bacillales*, *f_Bacillaceae,* and *g_Bacillus*. In the RYC group, the abundance of *f_norank_o__Clostridia_UCG-014*, *o_Clostridia_UCG-014*, *g_norank_f__norank_o__Clostridia_UCG-014*, *o_Clostridia_vadinBB60_group*, *g_norank_f__norank_o__Clostridia_vadinBB60_group,* and *f_norank_o__Clostridia_vadinBB60_group* was elevated. The BS + RYC group showed an increased abundance of *g_Ruminococcus_torques_group*, *g_norank_f__norank_o__norank_c_Clostridia*, *f_norank_o__norank_c_Clostridia*, *p_Desulfobacterota*, *c_Desulfovibrionia*, *f_Desulfovibrionaceae*, *o_Desulfovibrionales,* and *g_Bilophila*.

## 4. Discussion

Throughout the feeding trial in this study, broilers across all experimental groups were observed to exhibit comparable feed intake levels and similar daily weight gain trends. These observations suggest that the primary effects of RYC and BS supplementation are not driven by enhanced nutrient utilization for growth but rather by their regulatory roles in intestinal function. Specifically, RYC and BS were found to enhance intestinal barrier integrity and stimulate immune activity, which may incur physiological costs such as elevated immune resource allocation and increased intestinal mucus production. Consequently, these metabolic demands are likely to divert energy away from direct body protein deposition [12]. The improvements in intestinal health observed in yellow-feathered broilers following RYC and BS primarily involved enhanced villus morphology, upregulation of the *MUC2* gene, and optimized cecal microbiota composition. These findings are consistent with and corroborated by previous studies [22,27,36].

The structural integrity of intestinal tissue and intercellular junction complexes is fundamental to maintaining gut barrier homeostasis. In broilers, the development of the small intestinal mucosa directly influences digestion and absorption efficiency, thereby impacting overall health and performance [37]. The VH/CD ratio, a key indicator of intestinal development and absorptive capacity [38], was significantly increased in yellow-feathered broilers receiving combined RYC and BS supplementation. Findings from this research further verify the interactive effects of co-administration of RYC and BS. Notably, cosupplementation markedly elevated the jejunal VH/CD ratio, indicating synergism. Supporting our results, Xie et al. [39] demonstrated that fermented feed containing BS and yeast significantly improved the VH/CD ratio in Xuefeng black-bone chickens. The underlying mechanism may involve bacterial proteins and small peptides derived from RYC, which facilitate BS colonization in the jejunum while simultaneously stimulating villus growth and crypt cell proliferation [14,31]. Notably, dietary supplementation with BS alone did not significantly enhance intestinal VH and CD in the present study, consistent with the findings of Aliakbarpour et al. [40], who observed no significant improvement in jejunal villus architecture in 42-day-old broilers. This lack of effect may be attributed to age-dependent microbiota maturation, wherein established gut microbial communities competitively exclude exogenous BS colonization, thereby diminishing its promotive effects on intestinal villi [41].

TJPs regulate the function of the intestinal epithelial barrier and ensure its integrity by controlling permeability [42]. Interestingly, our results indicate that singular RYC administration downregulates ileal *OCLN* mRNA expression, demonstrating antagonism when coadministered with BS. In contrast, Kyoung et al. [14] found that the yeast cell wall components had no significant effect on ileal *OCLN* gene expression in broilers. These divergent outcomes may be attributed to multiple factors, including distinct yeast strains employed, disparities in supplementation levels, and differences in the breed and age of the broilers utilized. We speculate that RYC likely targets anti-inflammatory mechanisms as a primary mode of action, reallocating cellular resources to strengthen gut anti-inflammatory competence rather than fortifying the intestinal barrier via increased TJP mRNA expression. The mechanism underlying this effect warrants further investigation. Studies by Bilal et al. [43] revealed that dietary supplementation with *Bacillus pumilus* and *Bacillus subtilis* markedly elevated ileal *JAM2* and *TJP1* mRNA levels in broilers at 42 days of age. Consistent with prior findings, BS administration in this study yielded concordant *JAM2* and *TJP1* mRNA expression trends in ileal tissues.

*MUC2*, the primary structural component of intestinal mucus, plays a pivotal role in gut barrier defense by physically preventing pathogen adhesion and toxin penetration [44]. Findings from this study reveal that both RYC and BS significantly upregulate ileal *MUC2* mRNA expression yet exhibit no synergistic effect. This enhancement likely involves BS promoting goblet cell proliferation [40], while RYC provides the essential nutrients for epithelial cell metabolism and mucus production [45]. By strengthening the intestinal epithelial barrier, these mechanisms collectively enhance the integrity of the mucosal microstructure.

Immunoglobulin levels serve as critical biomarkers for evaluating humoral immune competence in poultry. The present study demonstrated that RYC supplementation significantly increased IgM concentrations in ileum tissue, consistent with Wang et al. [46], who report that yeast-derived products enhance jejunal immunoglobulins, and Kyoung et al. [14], who found that yeast cell walls increase the number of goblet cells in the duodenum of broilers. The underlying mechanism may involve β-glucans in RYC activating TLR2-mediated signaling in intestinal epithelial and antigen-presenting cells, subsequently promoting B lymphocyte differentiation into antibody-producing cells [47]. Findings from this study also reveal that dietary BS administration markedly elevated IgG levels in the ileum of yellow-feathered broilers, consistent with Dong et al.’s [48] report on serum IgG concentration in broilers fed diets containing *Bacillus subtilis* strain BYS2. Surprisingly, the combined supplementation of RYC and BS demonstrated an antagonistic effect on IgM in the ileal tissues of yellow-feathered broilers. We postulate that this observed antagonism may be attributable to organism-level dynamic immunoglobulin homeostasis. This phenomenon may reflect (i) the earlier emergence of IgM in primary immune responses, followed by (ii) IgG class switching during secondary responses [49]. Such sequential activation patterns could optimize humoral immunity across different stages of pathogen challenge, with IgM providing rapid first-line defense while IgG mediates sustained protection.

Cytokines play a crucial role in cell-mediated immunity, and their expression patterns reflect immune regulation. IFN-γ is a key Th1 cytokine, and its downregulation in our study suggests that RYC may help maintain immune homeostasis by promoting T regulatory cell differentiation. This aligns with Zhou et al. [50], who found that yeast cell wall polysaccharides suppress *IFNG* and *IL1B* upregulation in the ileum during *E. coli* challenge. The β-glucans in RYC likely contributes to this effect by enhancing *IL10* secretion, which inhibits IFN-γ production—consistent with Wang et al. [51], who observed similar immunomodulation with yeast hydrolysates in broilers. In addition, RYC contains yeast cell wall components (β-glucans and mannan oligosaccharides), which can reduce intestinal inflammation by adsorbing harmful bacteria [15]. Meanwhile, BS supplementation significantly increased ileal IL-1β levels, indicating its immune-stimulating properties, possibly due to transient inflammatory responses triggered by microbial colonization [18]. Khan et al. [52] reported similar findings, with BS elevating *IL1B* expression in the spleen and kidney. The combination of RYC and BS appears to establish a balanced regulatory model. While BS maintains baseline IL-1β (indicating immune activation), RYC counteracts excessive inflammation by scavenging reactive oxygen species via carotenoids [45,53].

Interestingly, while RYC alone reduced IL-1β protein levels, the BS + RYC group showed elevated *IL1B* mRNA expression. This discrepancy may reflect post-transcriptional regulation or compensatory feedback mechanisms. RYC could suppress IL-1β protein synthesis via NF-κB inhibition, whereas BS colonization transiently stimulates IL1B transcription in immune cells, consistent with Rajput et al. [54], who found that Saccharomyces boulardii and BS B10 upregulated IL1B in avian dendritic cells. Overall, BS appears to dominantly regulate IL1B mRNA levels in broilers, while RYC modulates downstream inflammatory responses. An interactive effect was observed for RYC and BS regarding IL-6 at the transcriptional level, while IL-6 cytokine levels in the ileum of yellow-feathered broilers remained unaffected. The underlying mechanisms merit future in-depth exploration.

The TLR4/NF-κB pathway is crucial for the regulation of the intestinal immune system [55]. Research conducted by Rajput et al. [56] demonstrates that *Saccharomyces boulardii* and *Bacillus subtilis* strain B10 modulate broiler gut immune responses through upregulation of TLR, MyD88, and NF-κB mRNA expression. This activation induces NF-κB nuclear translocation via the MyD88-dependent pathway, resulting in the upregulation of *NFKB1* mRNA expression in the ileum tissue, which subsequently leads to the upregulation of *TNFA* and *IL1B* mRNA expression [52]. These results align with the previous findings reported by Yang et al. [57]. In contrast, RYC treatment specifically upregulated *IL10* mRNA expression, suggesting its potential role in mitigating intestinal inflammation through enhanced anti-inflammatory responses and mucosal protection mechanisms [23]. While BS activated the NF-κB signaling pathway and enhanced inflammatory factors IL-1β, the β-glucan component of RYC simultaneously enhanced IL-10 expression through T cell-mediated pathways. Studies conducted by Bai et al. [58] demonstrate that yeast culture supplementation in the diet of pseudobagrus ussuriensis mitigates intestinal inflammation through regulation of the TLR2-MyD88-NF-κB signaling cascade. Together, these complementary actions establish a sophisticated immune balance regulatory network. This mechanism creates a balance between immune system activation and inflammation control, which helps prevent excessive immune damage while strengthening the intestinal mucosa’s defense [59].

The intestinal microbiota plays a pivotal role in modulating local immune responses and overall immune function in poultry [60]. In the present study, *Firmicutes* emerged as the dominant phylum in the cecal microbiota of yellow-feathered broilers, aligning with findings from Guo et al. [61]. Dietary supplementation with BS probiotics significantly increased the abundance of *Bacillus* spp. in the intestinal microbiota. This observation is consistent with previous research by Liu et al. [31], demonstrating that *Bacillus*-based probiotics in poultry feed yield similar microbial shifts. BS spores exhibit remarkable resilience, surviving gastric acid and bile salt stress before proliferating extensively in the posterior intestinal tract under favorable conditions [29].

RYC supplementation influenced the cecal microbial community structure, particularly enriching anaerobic *Clostridia* species such as *norank_f__norank_o__Clostridia_UCG-014* and *g_norank_f__norank_o__Clostridia_vadinBB60_group* [62]. The mannan oligosaccharides in RYC served as a selective carbon source for these *Clostridia*, promoting their colonization and metabolic activity [63]. These bacteria are critical for fiber degradation and butyrate synthesis, with elevated butyrate levels enhancing intestinal barrier integrity [64,65]. RYC jointly optimized the cecal bacterial community composition of yellow-feathered broilers by providing exclusive carbon sources, combining BS-suppressed pathogenic or competing bacteria, and the construction of an anaerobic microenvironment [25,66].

The BS-supplemented group exhibited significant enrichment of taxa classified under the *Bacillus* order, consistent with findings by Liu et al. [31], who reported that BS HC6 supplementation in white-feathered broilers enhanced cecal microbiota pathways linked to energy metabolism. In contrast, RYC supplementation prominently enriched the *Clostridia UCG-014* and *Clostridia vadinBB60* branches, both core members of the *Clostridia* class. Notably, the presence of unclassified microbial groups suggests that RYC may stimulate the proliferation of metabolically active but poorly characterized microbiota [36,67]. The specific interaction effect of RYC and BS co-addition showed significant enrichment of *p_Desulfobacterota, g_Ruminococcus_torques_group*, and *g_Bilophila*. The different experimental groups formed a characteristic flora structure. The BS group developed a *Bacillus*-dominated colony structure, while the BS + RYC group developed a *Desulfovibrio*-dominated colony structure. The CON group demonstrated distinct functional differentiation characteristics of the *Firmicutes* phylum, whereas the RYC group demonstrated those of the genus *Clostridia*. These structural differences likely modulate intestinal barrier function through multiple metabolic pathways, including short-chain fatty acid production, bile acid transformation, and sulfur cycling [68].

## 5. Conclusions

These findings highlight that dietary supplementation with RYC and BS primarily influences intestinal health and immune function rather than growth performance in yellow-feathered broilers. The combined use of RYC and BS may offer synergistic benefits for improving intestinal morphology. The combined use of RYC and BS revealed an antagonistic interaction, where the effects of one supplement were counteracted by the other in physical and chemical barriers and immune response. These findings highlight the complex interplay between RYC and BS in modulating gut health and immune responses, providing valuable insights for optimizing broiler nutrition and health management strategies in yellow-feathered broilers.

## Figures and Tables

**Table 1:** Composition and nutrient levels of the basal diet (%, as fed).

Items	1 to 28 Days of Age	29 to 56 Days of Age
Ingredients		
Maize	58.95	60.90
Soybean oil	3.00	3.00
Soybean meal	23.00	20.00
Cottonseed cake	6.00	6.00
Rape seed cake	5.00	6.00
Limestone	1.07	1.22
CaHPO_4_	1.90	1.80
NaCl	0.37	0.37
Choline	0.11	0.11
Vitamin premix ^1^	0.10	0.10
Mineral premix ^2^	0.50	0.50
Total	100.00	100.00
Nutrient levels		
ME (MJ/kg)	12.26	12.64
CP (%)	21.00	20.00
CF (%)	7.00	6.00
Ca (%)	1.05	0.95
P (%)	0.52	0.47
Met + Cys (%)	0.82	0.71

Abbreviations: Each kilogram of the diet contains one premix ^1^, which includes the following concentrations: vitamin A at 9000 IU, vitamin B1 at 3 mg, vitamin B2 at 8 mg, vitamin B6 at 4.4 mg, vitamin B12 at 0.012 mg, vitamin D3 at 3000 IU, vitamin E at 26 IU, vitamin K3 at 1.20 mg, D-pantothenic acid at 1 mg, nicotinic acid at 45 mg. Mineral premix ^2^ copper (Cu) at 10 mg, iron (Fe) at 100 mg, manganese (Mn) at 120 mg, zinc (Zn) at 108 mg, iodine (I) at 108 mg, and selenium (Se) at 0.35 mg. Metabolic energy (ME) was calculated based on data from the Chinese Raw Material Database on animal nutrition.

**Table 2:** Primers for RT–qPCR assessment of tissue RNA.

Gene Name	Primer Sequence (5′→3′)	Accession No.
*O* *CLN*	F: AGGTCTGCAACAGCATCACA	NM_205128.1
	R: ATGCCTTCCCAAAAAGCCCT	
*TJP* *1*	F: TATGCACAAGGAGGTCAGCC	XM_040680630.2
	R: TTGGCCGAAGCATTCCATCT	
*JAM2*	F: AGCCTCAAATGGGATTGGATT	XM_046907880.1
	R: CATCAACTTGCATTCGCTTCA	
*MUC2*	F: AAATGTATCTGTCGCCCCTCA	XM_046942297.1
	R: TGTCGCCATCCTTTATTGTTG	
*IL1* *B*	F: CAGCCTCAGCGAAGAGACCT	NM_204524.2
	R: ACTGTGGTGTGCTCAGAATCC	
*IL6*	F: AAATCCCTCCTCGCCAATCT	NM_204628.2
	R: CCCTCACGGTCTTCTCCATAAA	
*IL10*	F: AGGAGACGTTCGAGAAGATGGA	NM_001004414.4
	R: TCAGCAGGTACTCCTCGATGT	
*TNF* *A*	F: CTGTGGGGCGTGCAGTG	NM_204267.2
	R: GGCACAAAAGAGCTGATGGC	
*IFN* *G*	F: AGCTCCCGATGAACGACTTG	NM_205149.2
	R: TCCTCTGAGACTGGCTCCTT	
*MyD88*	F: CCTGGCTGTGCCTTCGGA	NM_001030962.5
	R: TCACCAAGTGCTGGATGCTA	
*TLR4*	F: TTCAGAACGGACTCTTGAGTGG	NM_001030693.2
	R: CAACCGAATAGTGGTGACGTTG	
*NF* *K* *B* *1*	F: GAAGGAATCGTACCGGGAACA	NM_001396396.1
	R: CTCAGAGGGCCTTGTGACAGTAA	
*β-actin*	F: GCCAACAGAGAGAAGATGACAC	NM_205518.2
	R: GTAACACCATCACCAGAGTCCA	

Abbreviations: F = forward primer, R = reverse primer. Occludin (*OCLN*), zonula occludin-1 (*TJP1*), junctional adhesion molecule-2 (*JAM2*), mucin-2 (*MUC2*), interleukin-1beta (*IL1B*), interleukin-6 (*IL6*), interleukin-10 (*IL10*), tumor necrosis factor-alpha (*TNFA*), interferon-γ (*IFNG*), myeloid differentiation factor 88 (*MyD88*), toll-like receptor 4 (*TLR4*), and nuclear transcription factor-κBp50 (*NFKB1*).

**Table 3:** Effects of feeding RYC and BS on the growth performance of yellow-feathered broilers from 1 to 56 days of age.

Items	CON		RYC		SEM	*p*-Value		
	CON	BS	CON	RYC + BS		RYC	BS	RYC × BS
ADFI (g/day)	96.05	95.05	94.80	95.12	3.013	0.543	0.929	0.928
ADG (g/day)	36.65	35.55	35.43	36.49	0.572	0.893	0.985	0.307
F/G	2.60	2.63	2.64	2.61	0.05	0.949	0.920	0.724
BW (g)	2122.14	2046.10	2055.90	2098.10	25.691	0.876	0.748	0.246

Abbreviations: Average daily feed intake (ADFI), average daily gain (ADG), feed conversion ratio (F/G), body weight (BW). Control group (CON), basal diet; *Bacillus subtilis* group (BS), basal diet + 5 × 10^9^ CFU/kg BS; *Rhodotorula* yeast culture group (RYC), basal diet + 5000 mg/kg RYC; BS + RYC group (BS + RYC), basal diet + 5 × 10^9^ CFU/kg BS + 5000 mg/kg RYC. SEM, standard error of the mean (*n* = 6).

**Table 4:** Effects of feeding RYC and BS on the intestinal morphology of yellow-feathered broilers.

Items		CON		RYC		SEM	*p*-Value		
		CON	BS	CON	RYC + BS		RYC	BS	RYC × BS
Duodenum	VH (μm)	1188.06	1247.31	1366.59	1248.56	35.89	0.239	0.245	0.688
	CD (μm)	73.13	66.22	76.91	69.67	1.58	0.175	0.947	0.020
	VH/CD	16.34	18.85	17.81	18.16	0.71	0.806	0.505	0.382
Jejunum	VH (μm)	954.70	1065.32	1011.16	1108.06	63.35	0.963	0.737	0.488
	CD (μm)	77.58	79.66	76.07	69.82	4.14	0.668	0.561	0.829
	VH/CD	12.31 ^b^	13.30 ^b^	13.23 ^b^	15.77 ^a^	0.45	0.200	0.016	0.013
Ileum	VH (μm)	707.28	789.69	711.66	726.35	48.62	0.769	0.798	0.675
	CD (μm)	71.43	65.10	62.66	58.99	2.19	0.748	0.101	0.249
	VH/CD	9.87	12.04	11.32	12.13	0.60	0.605	0.560	0.274

Abbreviations: VH (villus height); CD (crypt depth); the VH to CD ratio (VH/CD); control group (CON), basal diet; *Bacillus subtilis* group (BS), basal diet + 5 × 10^9^ CFU/kg BS; *Rhodotorula* yeast culture group (RYC), basal diet + 5000 mg/kg RYC; BS + RYC group (BS + RYC), basal diet + 5 × 10^9^ CFU/kg BS + 5000 mg/kg RYC. SEM, standard error of the mean (*n* = 6). ^a,b^ Means within a row with different superscripts differ significantly (*p* < 0.05).

**Table 5:** Effects of supplementation on RYC and BS on the expression regulation of tight junction-related proteins and *mucin2* genes in the ileum of yellow-feathered broilers.

Items	CON		RYC		SEM	*p*-Value		
	CON	BS	CON	RYC + BS		RYC	BS	RYC × BS
*O* *CLN*	1.00 ^b^	1.32 ^a^	1.01 ^b^	0.77 ^b^	0.06	0.012	0.696	0.009
*JAM2*	1.00 ^c^	2.72 ^a^	1.65 ^bc^	1.96 ^b^	0.17	0.816	<0.001	0.006
*TJP1*	1.00 ^c^	2.26 ^a^	1.38 ^bc^	1.79 ^ab^	0.13	0.809	<0.001	0.026
*MUC2*	1.00	1.87	1.42	2.22	0.11	0.009	<0.001	0.809

Abbreviations: Control group (CON), basal diet; *Bacillus subtilis* group (BS), basal diet + 5 × 10^9^ CFU/kg BS; *Rhodotorula* yeast culture group (RYC), basal diet + 5000 mg/kg RYC; BS + RYC group (BS + RYC), basal diet + 5 × 10^9^ CFU/kg BS + 5000 mg/kg RYC. SEM, standard error of the mean (*n* = 6). ^a,b,c^ Means within a row with different superscripts differ significantly (*p* < 0.05).

**Table 6:** Effects of feeding RYC and BS on the immune function of the ileum of yellow-feathered broilers.

Items	CON		RYC		SEM	*p*-Value		
	CON	BS	CON	BS + RYC		RYC	BS	RYC × BS
IgM (ng/mg prot.)	48.12 ^b^	48.88 ^b^	81.83 ^a^	47.80 ^b^	3.74	0.002	0.002	0.001
IgG (μg/mg prot.)	2.79	3.74	2.57	4.37	0.22	0.565	<0.001	0.235
sIgA (pg/mg prot.)	134.34	180.53	166.91	181.71	9.47	0.370	0.113	0.404
TNF-α (pg/mg prot.)	8.75	10.43	10.21	10.97	0.47	0.299	0.209	0.633
IFN-γ (pg/mg prot.)	22.25	21.97	15.34	20.87	1.03	0.041	0.166	0.128
IL-1β (pg/mg prot.)	11.55	23.94	11.31	17.82	1.27	0.037	<0.001	0.052
IL-6 (pg/mg prot.)	12.63	12.92	11.92	13.02	0.49	0.773	0.508	0.699
IL-10 (pg/mg prot.)	11.93	12.43	11.10	12.59	0.47	0.737	0.323	0.621

Abbreviations: Control group (CON), basal diet; *Bacillus subtilis* group (BS), basal diet + 5 × 10^9^ CFU/kg BS; *Rhodotorula* yeast culture group (RYC), basal diet + 5000 mg/kg RYC; BS + RYC group (BS + RYC), basal diet + 5 × 10^9^ CFU/kg BS + 5000 mg/kg RYC. SEM, standard error of the mean (*n* = 6). ^a,b^ Means within a row with different superscripts differ significantly (*p* < 0.05). Prot. indicates the total protein concentration as determined by the BCA method.

**Table 7:** Effects of RYC and BS supplementation on ileal immunity-related gene expression in yellow-feathered broilers.

Items	CON		RYC		SEM	*p*-Value		
	CON	BS	CON	BS + RYC		RYC	BS	RYC × BS
*TNF* *A*	1.00	1.96	1.70	2.36	0.17	0.074	0.011	0.608
*IFN* *G*	1.00	1.11	1.09	1.35	0.06	0.154	0.112	0.513
*IL1* *B*	1.00	1.69	0.86	1.61	0.06	0.791	<0.001	0.785
*IL6*	1.00	1.99	1.45	1.86	0.16	0.601	0.341	0.031
*IL10*	1.00	1.30	2.24	2.58	0.22	0.004	0.417	0.962
*TLR4*	1.00	0.71	0.97	0.87	0.10	0.761	0.358	0.639
*MyD88*	1.00	1.18	1.23	1.68	0.09	0.038	0.067	0.421
*NFΚ* *B1*	1.00	1.53	1.23	1.41	0.06	0.776	0.001	0.379

Abbreviations: Control group (CON), basal diet; *Bacillus subtilis* group (BS), basal diet + 5 × 10^9^ CFU/kg BS; *Rhodotorula* yeast culture group (RYC), basal diet + 5000 mg/kg RYC; BS + RYC group (BS + RYC), basal diet + 5 × 10^9^ CFU/kg BS + 5000 mg/kg RYC. SEM, standard error of the mean (*n* = 6).

## Data Availability

Data are available on request owing to restrictions. 16S rRNA sequencing data are available under NCBI BioProject PRJNA1199219 (accessible after 1 September 2025). All other datasets, including growth performance and so on, are available from the corresponding author upon reasonable request.

## References

[1] Liu Y., Tang Y., Mei H., Liu Z., Li Z., Ma X., Luo Z., Huang W., Li Y., Yu M. (2024). Feeding citrus pomace fermented with combined probiotics improves growth performance, meat quality, fatty acid profile, and antioxidant capacity in yellow-feathered broilers. Front. Vet. Sci..

[2] Fang X., Ye H., Zhang S., Guo L., Xu Y., Zhang D., Nie Q. (2023). Investigation of potential genetic factors for growth traits in yellow-feather broilers using weighted single-step genome-wide association study. Poult. Sci..

[3] Gao L., Liu C., Wu J., Cui Y., Zhang M., Bi C., Shan A., Dou X. (2025). EGCG improve meat quality, restore lipid metabolism disorder and regulate intestinal flora in high-fat fed broilers. Poult. Sci..

[4] Salvo Romero E., Alonso Cotoner C., Pardo Camacho C., Casado Bedmar M., Vicario M. (2015). The intestinal barrier function and its involvement in digestive disease. Rev. Esp. Enferm. Dig..

[5] Song M., Ren C., Liu Y., Ye X., Wang Y., Xie J., Zhao F. (2024). Comparison of the characteristics of small intestinal fluid from white-feathered and yellow-feathered broilers. Poult. Sci..

[6] Flemming S., Luissint A.C., Kusters D.H.M., Raya-Sandino A., Fan S., Zhou D.W., Hasegawa M., Garcia-Hernandez V., García A.J., Parkos C.A. (2020). Desmocollin-2 promotes intestinal mucosal repair by controlling integrin-dependent cell adhesion and migration. Mol. Biol. Cell.

[7] Yang L., Liu G., Lian K., Qiao Y., Zhang B., Zhu X., Luo Y., Shang Y., Gu X.L. (2019). Dietary leonurine hydrochloride supplementation attenuates lipopolysaccharide challenge-induced intestinal inflammation and barrier dysfunction by inhibiting the NF-κB/MAPK signaling pathway in broilers. J. Anim. Sci..

[8] Latue P.E., Ariyadi B., Kurniawati A., Al Anas M. (2025). Positive effect of fermented sorghum on productivity, jejunal histomorphology, and tight junction gene expression in broiler chickens. Poult. Sci..

[9] Jha R., Das R., Oak S., Mishra P. (2020). Probiotics (Direct-Fed Microbials) in Poultry Nutrition and Their Effects on Nutrient Utilization, Growth and Laying Performance, and Gut Health: A Systematic Review. Animals.

[10] Sjofjan O., Adli D.N., Harahap R.P., Jayanegara A., Utama D.T., Seruni A.P. (2021). The effects of lactic acid bacteria and yeasts as probiotics on the growth performance, relative organ weight, blood parameters, and immune responses of broiler: A meta-analysis. F1000Research.

[11] Bilal R.M., Hassan F.U., Saeed M., Rafeeq M., Zahra N., Fraz A., Saeed S., Khan M.A., Mahgoub H.A.M., Farag M.R. (2023). Role of Yeast and Yeast-Derived Products as Feed Additives in Broiler Nutrition. Anim. Biotechnol..

[12] Wu C., Yang Z., Song C., Liang C., Li H., Chen W., Lin W., Xie Q. (2018). Effects of dietary yeast nucleotides supplementation on intestinal barrier function, intestinal microbiota, and humoral immunity in specific pathogen-free chickens. Poult. Sci..

[13] Johnson C.N., Hashim M.M., Bailey C.A., Byrd J.A., Kogut M.H., Arsenault R.J. (2020). Feeding of yeast cell wall extracts during a necrotic enteritis challenge enhances cell growth, survival and immune signaling in the jejunum of broiler chickens. Poult. Sci..

[14] Kyoung H., Kim E., Cho J.H., Lee H., Kim Y., Park K.I., Kim H.B., Song M. (2023). Dietary yeast cell wall enhanced intestinal health of broiler chickens by modulating intestinal integrity, immune responses, and microbiota. Poult. Sci..

[15] Tapingkae W., Srinual O., Lumsangkul C., Doan H.V., Chiang H.I., Manowattana A., Boonchuay P., Chaiyaso T. (2022). Industrial-Scale Production of Mycotoxin Binder from the Red Yeast *Sporidiobolus pararoseus* KM281507. J. Fungi.

[16] Li Z., Li C., Cheng P., Yu G. (2022). *Rhodotorula mucilaginosa*-alternative sources of natural carotenoids, lipids, and enzymes for industrial use. Heliyon.

[17] Wickramasuriya S.S., Park I., Lee Y., Kim W.H., Przybyszewski C., Gay C.G., van Oosterwijk J.G., Lillehoj H.S. (2021). Oral Deliv-ery of *Bacillus subtilis* Expressing Chicken NK-2 Peptide Protects Against Eimeria acervulina Infection in Broiler Chickens. Front. Vet. Sci..

[18] Yang J., Huang K., Wang J., Wu D., Liu Z., Yu P., Wei Z., Chen F. (2021). Combined Use of *Bacillus subtilis* yb-114,246 and *Bacillus licheniformis* yb-214,245 Improves Body Growth Performance of Chinese Huainan Partridge Shank Chickens by Enhancing Intestinal Digestive Profiles. Probiotics Antimicrob. Proteins.

[19] Park I., Lee Y., Goo D., Zimmerman N.P., Smith A.H., Rehberger T., Lillehoj H.S. (2020). The effects of dietary *Bacillus subtilis* supplementation, as an alternative to antibiotics, on growth performance, intestinal immunity, and epithelial barrier integrity in broiler chickens infected with Eimeria maxima. Poult. Sci..

[20] Qiu K., Li C.L., Wang J., Qi G.H., Gao J., Zhang H.J., Wu S.G. (2021). Effects of Dietary Supplementation with *Bacillus subtilis*, as an Alternative to Antibiotics, on Growth Performance, Serum Immunity, and Intestinal Health in Broiler Chickens. Front. Nutr..

[21] Ningsih N., Respati A.N., Astuti D., Triswanto T., Purnamayanti L., Yano A.A., Putra R.P., Jayanegara A., Ratriyanto A., Irawan A. (2023). Efficacy of *Bacillus subtilis* to replace in-feed antibiotics of broiler chickens under necrotic enteritis-challenged experiments: A systematic review and meta-analysis. Poult. Sci..

[22] Yang J., Wang J., Huang K., Liu Q., Guo L., Xu X., Zhang H., Zhu M. (2021). Selenium-enriched *Bacillus subtilis* yb-114246 improved growth and immunity of broiler chickens through modified ileal bacterial composition. Sci. Rep..

[23] Fazelnia K., Fakhraei J., Yarahmadi H.M., Amini K. (2021). Dietary Supplementation of Potential Probiotics *Bacillus subtilis*, *Bacillus licheniformis*, and *Saccharomyces cerevisiae* and *Synbiotic* Improves Growth Performance and Immune Responses by Modulation in Intestinal System in Broiler Chicks Challenged with Salmonella Typhimurium. Probiotics Antimicrob. Proteins.

[24] Cheng Y.H., Zhang N., Han J.C., Chang C.W., Hsiao F.S., Yu Y.H. (2018). Optimization of surfactin production from *Bacillus subtilis* in fermentation and its effects on *Clostridium perfringens*-induced necrotic enteritis and growth performance in broilers. J. Anim. Physiol. Anim. Nutr..

[25] Zhao Y., Guo L., Xia Y., Zhuang X., Chu W. (2019). Isolation, Identification of Carotenoid-Producing *Rhodotorula* sp. from Marine Environment and Optimization for Carotenoid Production. Mar. Drugs.

[26] Maina A.N., Schulze H., Kiarie E.G. (2024). Response of broiler breeder pullets when fed hydrolyzed whole yeast from placement to 22 wk of age. Poult. Sci..

[27] Selionova M.I., Trukhachev V.I., Zagarin A.Y., Kulikov E.I., Belyaeva N.P. (2025). Effects of Dietary Supplementation Using Phytobiotics with Different Functional Properties on Expression of Immunity Genes, Intestinal Histology, Growth, and Meat Productivity of Broiler Chickens. Vet. Sci..

[28] Sun Z., Wang T., Demelash N., Zheng S., Zhao W., Chen X., Zhen Y., Qin G. (2019). Effect of Yeast Culture (*Saccharomyces cerevisiae*) on Broilers: A Preliminary Study on the Effective Components of Yeast Culture. Animals.

[29] Wang J., Ishfaq M., Miao Y., Liu Z., Hao M., Wang C., Wang J., Chen X. (2022). Dietary administration of *Bacillus subtilis* KC1 improves growth performance, immune response, heat stress tolerance, and disease resistance of broiler chickens. Poult. Sci..

[30] Ministry of Agriculture and Rural Affairs of China (2020). Nutrient Requirements of Yellow-Feathered Broilers (NY/T 3645-2020).

[31] Liu S., Xiao G., Wang Q., Zhang Q., Tian J., Li W., Gong L. (2023). Effects of Dietary *Bacillus subtilis* HC6 on Growth Performance, Antioxidant Capacity, Immunity, and Intestinal Health in Broilers. Animals.

[32] Ji Y., Liu X., Lv H., Guo Y., Nie W. (2024). Effects of *Lonicerae flos* and Turmeric extracts on growth performance and intestinal health of yellow-feathered broilers. Poult. Sci..

[33] Livak K.J., Schmittgen T.D. (2001). Analysis of relative gene expression data using real-time quantitative PCR and the 2(-Delta Delta C(T)) Method. Methods.

[34] Bolyen E., Rideout J.R., Dillon M.R., Bokulich N.A., Abnet C.C., Al-Ghalith G.A., Alexander H., Alm E.J., Arumugam M., Asnicar F. (2019). Asnicar F Reproducible, interactive, scalable and extensible microbiome data science using QIIME 2. Nat. Biotechnol..

[35] IBM Corp (2012). IBM SPSS Statistics for Windows (Version 21.0) [Computer Software].

[36] Sun Z., Wang T., Aschalew N.D., Zhao W., Chen X., Zhang X.F., Zhen Y.G., Qin G.X. (2020). Effects of yeast cultures with different fermentation times on the growth performance, caecal microbial community and metabolite profile of broilers. J. Anim. Physiol. Anim. Nutr..

[37] Peterson L.W., Artis D. (2014). Intestinal epithelial cells: Regulators of barrier function and immune homeostasis. Nat. Rev. Immunol..

[38] Marquis V., Schulthess J., Molist F., Santos R.R. (2025). Effect of a Yeast β-Glucan on the Performance, Intestinal Integrity, and Liver Function of Broiler Chickens Fed a Diet Naturally Contaminated with *Fusarium* Mycotoxins. Toxins.

[39] Xie Y., Liu J., Wang H., Luo J., Chen T., Xi Q., Zhang Y., Sun J. (2020). Effects of fermented feeds and ginseng polysaccharides on the intestinal morphology and microbiota composition of Xuefeng black-bone chicken. PLoS ONE.

[40] Aliakbarpour H.R., Chamani M., Rahimi G., Sadeghi A.A., Qujeq D. (2012). The *Bacillus subtilis* and Lactic Acid Bacteria Probiotics Influences Intestinal Mucin Gene Expression, Histomorphology and Growth Performance in Broilers. Asian-Australas. J. Anim. Sci..

[41] Yang J., Zhan K., Zhang M. (2020). Effects of the Use of a Combination of Two Bacillus Species on Performance, Egg Quality, Small Intestinal Mucosal Morphology, and Cecal Microbiota Profile in Aging Laying Hens. Probiotics Antimicrob. Proteins.

[42] Awad W.A., Hess C., Hess M. (2017). Enteric Pathogens and Their Toxin-Induced Disruption of the Intestinal Barrier Through Alteration of Tight Junctions in Chickens. Toxins.

[43] Bilal M., Si W., Barbe F., Chevaux E., Sienkiewicz O., Zhao X. (2021). Effects of novel probiotic strains of *Bacillus pumilus* and *Bacillus subtilis* on production, gut health, and immunity of broiler chickens raised under suboptimal conditions. Poult. Sci..

[44] Qiao Y., Liu C., Guo Y., Zhang W., Guo W., Oleksandr K., Wang Z. (2022). Polysaccharides derived from *Astragalus membranaceus* and *Glycyrrhiza uralensis* improve growth performance of broilers by enhancing intestinal health and modulating gut microbiota. Poult. Sci..

[45] Hui J., Li L., Li R., Wu M., Yang Y., Wang J., Fan Y., Zheng X. (2020). Effects of supplementation with β-carotene on the growth performance and intestinal mucosal barriers in layer-type cockerels. Anim. Sci. J..

[46] Wang T., Cheng K., Yu C.Y., Li Q.M., Tong Y.C., Wang C., Yang Z.B., Wang T. (2021). Effects of a yeast-derived product on growth performance, antioxidant capacity, and immune function of broilers. Poult. Sci..

[47] Martín D., Ordás M.C., Morel E., Nuñez-Ortiz N., Díaz-Rosales P., Vicente-Gil S., Zarza C., Jensen L., Tafalla C. (2024). Effect of β-glucans on rainbow trout (*Oncorhynchus mykiss*) IgM+ B cells. Fish Shellfish Immunol..

[48] Dong Y., Li R., Liu Y., Ma L., Zha J., Qiao X., Chai T., Wu B. (2020). Benefit of Dietary Supplementation with *Bacillus subtilis* BYS2 on Growth Performance, Immune Response, and Disease Resistance of Broilers. Probiotics Antimicrob. Proteins.

[49] Yang T.C., Li H., Huang G.N., Wang S.Y. (2007). Detection of IgM and IgG complexes provides new insight into immune regulation of patients with malignancies: A randomized controlled trial. Int. Immunopharmacol..

[50] Zhou J., Fu Y., Qi G., Dai J., Zhang H., Wang J., Wu S. (2023). Yeast cell-wall polysaccharides improve immunity and attenuate inflammatory response via modulating gut microbiota in LPS-challenged laying hens. Int. J. Biol. Macromol..

[51] Wang T., Cheng K., Li Q., Wang T. (2022). Effects of yeast hydrolysate supplementation on intestinal morphology, barrier, and anti-inflammatory functions of broilers. Anim. Biosci..

[52] Khan S., Khalid A., Yang R., Khalid F., Zahid M.H., Liu H., Zhang Y., Wang Z. (2024). Effect of *Bacillus subtilis* Supplemented Diet on Broiler’s Intestinal Microbiota and TLRs Gene Expression. Probiotics Antimicrob. Proteins.

[53] Wang M.Y., Zhang Y., Tong Y.X., Guo P.T., Zhang J., Wang C.K., Gao Y.Y. (2022). Effects of lutein on jejunal mucosal barrier function and inflammatory responses in lipopolysaccharide-challenged yellow-feather broilers. Poult. Sci..

[54] Rajput I.R., Hussain A., Li Y.L., Zhang X., Xu X., Long M.Y., You D.Y., Li W.F. (2014). Saccharomyces boulardii and *Bacillus subtilis* B10 modulate TLRs mediated signaling to induce immunity by chicken BMDCs. J. Cell. Biochem..

[55] Bi Y., Wei H., Chai Y., Wang H., Xue Q., Li J. (2024). Intermittent mild cold acclimation ameliorates intestinal inflammation and immune dysfunction in acute cold-stressed broilers by regulating the TLR4/MyD88/NF-κB pathway. Poult. Sci..

[56] Rajput I.R., Ying H., Yajing S., Arain M.A., Weifen L., Ping L., Bloch D.M., Wenhua L. (2017). *Saccharomyces boulardii* and *Bacillus subtilis* B10 modulate TLRs and cytokines expression patterns in jejunum and ileum of broilers. PLoS ONE.

[57] Yang J., Wang J., Huang K., Zhu M., Liu Q., Liu G., Chen F., Zhang H., Qin S. (2021). Selenium enriched *Bacillus subtilis yb-1*114246 activated the TLR2-NF-κB1 signaling pathway to regulate chicken intestinal β-defensin 1 expression. Food Funct..

[58] Bai Y., Liao Y., Song Y., Wang J., Deng X., Luan L., An N., Zhou W., Liang T., Yang Y. (2022). Dietary yeast culture alleviates intestinal-hepatic damage related to TLR2-MyD88-NF-κB signaling pathway and antioxidant capability in *Pseudobagrus ussuriensis*. Fish Shellfish Immunol..

[59] Tarasiuk-Zawadzka A., Fichna J. (2025). Interaction between nutritional factors and the enteric nervous system in inflammatory bowel diseases. J. Nutr. Biochem..

[60] Yue Y., Luasiri P., Li J., Laosam P., Sangsawad P. (2024). Research advancements on the diversity and host interaction of gut microbiota in chickens. Front. Vet. Sci..

[61] Guo P., Lin S., Lin Q., Wei S., Ye D., Liu J. (2023). The digestive tract histology and geographical distribution of gastrointestinal microbiota in yellow-feather broilers. Poult. Sci..

[62] Rodrigues D.R., Briggs W., Duff A., Chasser K., Murugesan R., Pender C., Ramirez S., Valenzuela L., Bielke L.R. (2020). Comparative effectiveness of probiotic-based formulations on cecal microbiota modulation in broilers. PLoS ONE.

[63] Oba S., Washida K., Shimada Y., Sunagawa T., Tanihiro R., Sugiyama H., Nakamura Y. (2020). Yeast mannan increases *Bacteroides thetaiotaomicron* abundance and suppresses putrefactive compound production in in vitro fecal microbiota fermentation. Biosci. Biotechnol. Biochem..

[64] Liu L., Li Q., Yang Y., Guo A. (2021). Biological Function of Short-Chain Fatty Acids and Its Regulation on Intestinal Health of Poultry. Front. Vet. Sci..

[65] Melaku M., Zhong R., Han H., Wan F., Yi B., Zhang H. (2021). Butyric and Citric Acids and Their Salts in Poultry Nutrition: Effects on Gut Health and Intestinal Microbiota. Int. J. Mol. Sci..

[66] Oketch E.O., Yu M., Hong J.S., Chaturanga N.C., Seo E., Lee H., Hermes R.G., Smeets N., Taechavasonyoo A., Kirwan S. (2024). Laying hen responses to multi-strain *Bacillus*-based probiotic supplementation from 25 to 37 weeks of age. Anim. Biosci..

[67] Wang J., Zheng Z., Yang H., Chen J., Xiao Y., Ji X., Zhang Z., He H., Ding B., Tang B. (2022). Effect of β-1,3/1,6-glucan on gut microbiota of yellow-feathered broilers. AMB Express.

[68] Qiu M., Hu J., Peng H., Li B., Xu J., Song X., Yu C., Zhang Z., Du X., Bu G. (2022). Research Note: The gut microbiota varies with dietary fiber levels in broilers. Poult. Sci..

